# Monocytes and macrophages: emerging mechanisms and novel therapeutic targets in pulmonary fibrosis

**DOI:** 10.1152/ajpcell.00302.2023

**Published:** 2023-09-11

**Authors:** Carole Y. Perrot, Theodoros Karampitsakos, Jose D. Herazo-Maya

**Affiliations:** Ubben Center for Pulmonary Fibrosis Research, Division of Pulmonary, Critical Care and Sleep Medicine, Department of Internal Medicine, Morsani College of Medicine, University of South Florida, Tampa, Florida, United States

**Keywords:** immunity, macrophages, monocytes, pathogenesis, pulmonary fibrosis

## Abstract

Pulmonary fibrosis results from a plethora of abnormal pathogenetic events. In idiopathic pulmonary fibrosis (IPF), inhalational, environmental, or occupational exposures in genetically and epigenetically predisposed individuals trigger recurrent cycles of alveolar epithelial cell injury, activation of coagulation pathways, chemoattraction, and differentiation of monocytes into monocyte-derived alveolar macrophages (Mo-AMs). When these events happen intermittently and repeatedly throughout the individual’s life cycle, the wound repair process becomes aberrant leading to bronchiolization of distal air spaces, fibroblast accumulation, extracellular matrix deposition, and loss of the alveolar-capillary architecture. The role of immune dysregulation in IPF pathogenesis and progression has been underscored in the past mainly after the disappointing results of immunosuppressant use in IPF patients; however, recent reports highlighting the prognostic and mechanistic roles of monocytes and Mo-AMs revived the interest in immune dysregulation in IPF. In this review, we will discuss the role of these cells in the onset and progression of IPF, as well as potential targeted therapies.

## Graphical abstract

**Figure:**
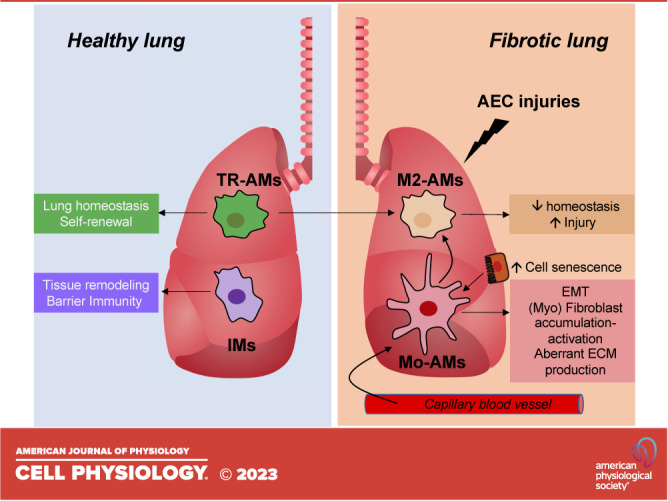

## INTRODUCTION

Pulmonary fibrosis (PF) represents the end stage of several, heterogeneous interstitial lung diseases (1, 2). More than 200 causes of PF have been described including autoimmune diseases, genetic disorders, drugs, and environmental and occupational exposure to injurious stimuli. If none of these causes is identified, patients with usual interstitial pneumonia pattern are diagnosed with idiopathic pulmonary fibrosis (IPF) (3).

A plethora of abnormal pathogenetic events happening intermittently and repeatedly over time lead to IPF development in genetically and epigenetically predisposed individuals of advanced age. The disease initiates with alveolar epithelial cell injury, activation of coagulation pathways and chemoattraction, and differentiation of monocytes into monocyte-derived alveolar macrophages (Mo-AMs). When these events become cyclical and recur over time, key cells participating in this process become senescent and develop abnormal metabolism and processes leading to aberrant wound repair, characterized by fibroblast accumulation, extracellular matrix deposition, bronchiolization of distal air spaces, and loss of the alveolar-capillary architecture (4–21). As the disease progresses, the immune system becomes dysregulated, and exaggerated immune responses lead to increased IPF mortality (22–24). Recent reports demonstrated that increased monocyte counts and monocyte-specific genes as well as decreased T-cell subpopulations and T-cell costimulatory pathways have been associated with increased risk of IPF mortality and poor disease outcomes (23–29). The aforementioned, coupled with experimental evidence showing that newly recruited monocyte-derived macrophages contribute to lung fibrosis pathogenesis and progression after alveolar epithelium injury through the secretion of several profibrotic mediators, led to a revived interest in the role of immune dysregulation in IPF and highlights the importance of monocytes and macrophages as key immune cells participating in the process of aberrant lung injury and repair (30–32). In this review, we will discuss the emerging role of monocytes and macrophages in PF pathogenesis and progression and highlight mechanistic insights.

## POPULATIONS OF MACROPHAGES IN THE HEALTHY LUNG

**Figure 1.**
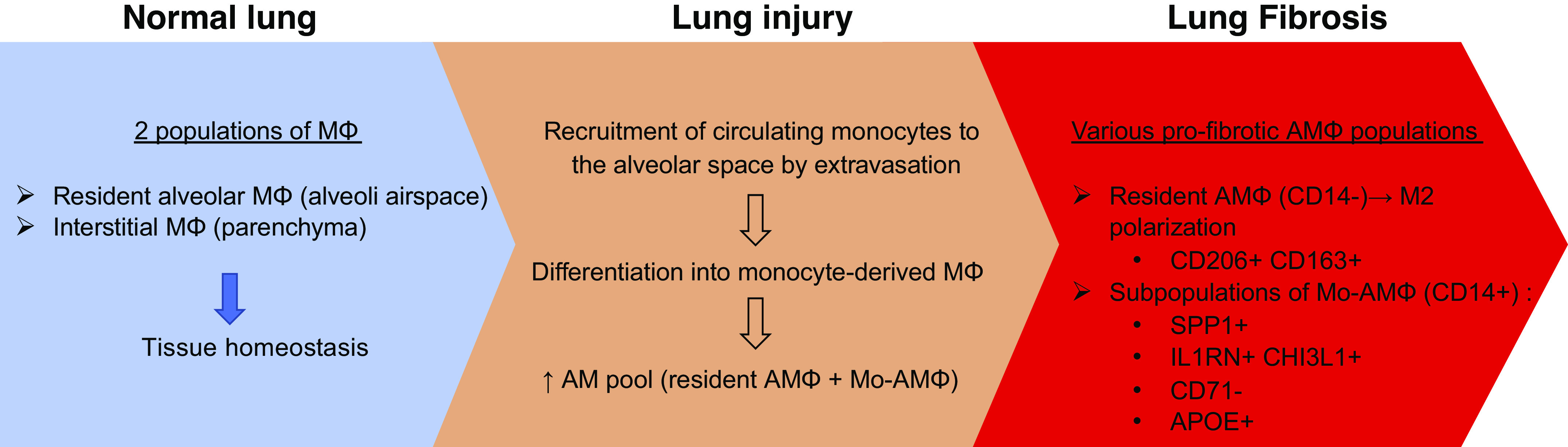
Emergence of monocyte (M) and alveolar macrophage (AM) populations in pulmonary fibrosis. Mo-AM, monocyte-derived alveolar macrophage.

Macrophages, the most abundant immune cell type in healthy lungs, can be classified into two main populations: alveolar macrophages (AMs) and interstitial macrophages (IMs). AMs reside in the lumen of distal lung alveoli as well as in the interalveolar septum in proximity to pneumocytes. AMs are the primary resident innate immune cells in the lungs and exert a critical role in tissue homeostasis: they recognize and clear inhaled pathogens and debris, catabolize surfactants, and orchestrate the initiation and resolution of inflammation (33). Like other tissue-resident macrophages, AMs represent a self-renewing population that originates from embryonic progenitors and requires minimal input from peripheral blood monocytes in normal conditions (34–36). Following lung injury, circulating monocytes extravasate from blood vessels and infiltrate the tissue where they differentiate into Mo-AMs, further enriching the pool of resident AMs (37) (Fig. 1).

Interstitial macrophages (IMs) are located in the lung interstitium. Compared to AMs, IMs are smaller, with a size and morphology similar to blood monocytes. Little is known about the potential proinflammatory activities of IMs, and most of the studies have focused on their potential immunoregulatory properties. Indeed, mouse and human IMs have been shown to express the immunosuppressive cytokine IL-10 in the steady state (38). IMs are also involved in tissue remodeling and participate in barrier immunity as antigen-presenting cells (33). An additional population of macrophages called intravascular macrophages, attached to the capillary endothelium, has also been described in humans and other mammals. However, their role remains poorly documented (33, 39).

## THE MACROPHAGE POLARIZATION PARADIGM

Macrophages are cells with a high degree of plasticity and display a remarkable capacity to switch from one phenotype to another (40). Beyond the AM/IM macrophage subtypes, polarized activation of macrophages distinguishes classically activated (M1) and alternatively activated (M2) macrophages, the latter being divided into M2a, M2b, M2c, and M2d subcategories (40). The central suggested concept is that M1 macrophages suppress, while M2 macrophages promote, fibrosis and aberrant wound healing (31).

M1 macrophage polarization occurs upon exposure to inflammatory molecules [e.g., T helper (Th)1 cell-secreted cytokines, including interferon-γ (IFN-γ) and tumor necrosis factor-α (TNF-α)], pathogen-associated molecular patterns (e.g., lipopolysaccharide), and damage-associated molecular patterns (e.g., HMGB1, heat shock proteins, ATP, fetuin-A, and oxidized low-density-lipoprotein) (41–45).

M1 macrophage markers include CD80, CD86, inducible nitric oxide synthase (iNOS), major histocompatibility complex (MHC)-II, Toll-like receptor (TLR)-2, and TLR-4. These cells release various cytokines and chemokines, such as TNF-α, IL-1α, IL-1β, IL-6, IL-12, CXC motif chemokine ligand (CXCL)9, CXCL10, and CXCL11, which exert positive feedback on unpolarized macrophages (41, 43, 46). NF-κB, STAT1, IRF3, and IRF5 signaling pathways are the main pathways involved in the regulation of an M1 polarization and transcriptional program, resulting in M1 powerful cytotoxic activities (41, 47).

M2 polarization occurs in response to downstream signals of cytokines such as IL-4, IL-13, IL-10, IL-33, and transforming growth factor-β (TGF-β) (31, 48). IL-4 and IL-13 directly induce M2 macrophage activation, whereas other cytokines (such as IL-33 and IL-25) amplify M2 macrophage activation by producing Th2 cytokines (49). M2 macrophages resolve inflammation via upregulation of anti-inflammatory mediators, such as IL-10, TGF-β, IL1-R type II, and IL-1Ra, and recruit Th2, Tregs, as well as eosinophils and basophils through C-C motif chemokine ligand (CCL)17, CCL18, CCL22, and CCL24 (50). They express markers such as the scavenger receptors CD206 and CD163, as well as fibrosis-related proteins like arginase-1, FIZZ1, chitinase 3 like-1 and -2 (YKL-40 in humans), CCL18, and IL-1β, which contribute to fibroblast activation and ECM accumulation in the lung (51–53). The M2 phenotype is notably driven and maintained by STAT3 and -6, peroxisome proliferator-activated receptor (PPAR)δ, and PPARγ signaling pathway (41, 49). Mechanistically, IL-4 stimulation leads to STAT6 phosphorylation, which activates the transcription of profibrotic target genes (54). KLF4, a member of the Krüppel-like factor family, has been shown to induce monocyte differentiation in vivo and to suppress inflammatory gene expression in macrophages, inducing an M2 phenotype (55, 56). Conversely, KLF4-deficient macrophages demonstrated increased proinflammatory gene expression and enhanced bactericidal activity, two features of M1-macrophages (56).

Epigenetic regulation of chromatin activity has also emerged as an important factor in polarized activation. Histone acetylation and methylation influence M1/M2 polarization. For instance, histone deacetylase 3 (HDAC3) has been shown to promote M1 polarization, and HDAC3^-/-^ macrophages skew toward the M2 phenotype(57). In contrast, IL-4-induced expression of H3K27 demethylase Jumonji domain containing-protein 3 (Jmjd3) results in the upregulation of M2 genes and subsequently M2 polarization (58, 59). Posttranslational epigenetic regulation controlled by micro-RNAs (miRNA) represents an additional regulatory mechanism of macrophage polarization. For instance, miR-155 supports M1-driven inflammation following stimulation by TLR ligands, while it inhibits the M2 program by interfering with IL-13 and TGF-β signaling (14, 41, 60–62). miR-125b also enhances M1 polarization while miR-223, -124, and -125a-5p and Let-c promote a M2 program (63).

## MONOCYTES AND MACROPHAGES IN PULMONARY FIBROSIS

### Circulating Monocytes and Monocyte-Specific Genes as Biomarkers of PF Progression and Mortality

The contribution of monocytes to lung fibrosis is relatively poorly documented, compared to macrophages. However, the research interest in monocytes has been recently revived following translational studies reporting that a peripheral blood 52-gene expression signature classified patients with IPF into low- or high-risk groups for mortality (1, 22, 23). Cellular deconvolution of this 52-gene signature showed that monocytes represent the cellular source of the high-risk profile (23). This finding fueled further studies for the prognostic role of monocyte counts as markers of IPF mortality and decreased survival in other fibrotic disorders. Both pooled analysis of clinical trials and real-life data have demonstrated that increased monocyte counts are indeed linked to increased risk of IPF progression, hospitalization, and mortality (1, 24–26, 64).

Translational research studies showed that monocytes in patients with IPF are phenotypically different from age-matched controls, exhibiting a primed type I interferon pathway and markedly high serum levels of CSF-1, CCL-2, and IL-6 levels (65). Authors also performed single-cell RNA-sequencing analysis of cell suspensions from IPF lung tissues and healthy donors and showed a progression in transcriptomic states from monocytes (CD14^+^CD206^neg/lo^CD69^neg/lo^) to early transitional macrophages (CD14^+^CD206^lo^, CD68^lo^), to later transitional macrophages (CD14^+^CD206^lo^, CD68^mid-hi^), to lung macrophages (CD206^mid-hi^CD68^mid-hi^CD14^neg/lo^) (65).

In addition to translational studies in IPF and other fibrotic disorders, murine models of PF demonstrated that both newly recruited monocyte-derived lung macrophages and long-lived lung resident AMs contribute to the progression of PF (33, 37, 66). Evren and colleagues (33) showed that extravasating CD14^+^ monocytes give rise to AM and IMs. The authors used MISTRG, a humanized mouse model, and adoptively transferred purified blood monocytes (CD14^+^CD16^–^ monocytes) subsets into adult MISTRG mice and generated human lung macrophages with an AM phenotype (CD45^+^HLA-DR^+^CD11b^+^CD206^+^CD169^+^) 3 weeks posttransfer. In contrast, they showed that nonclassical CD14^lo^CD16^+^ monocytes were confined to the lung vasculature and gave rise to a distinct population of pulmonary intravascular macrophages. Misharin and colleagues (67) showed that 5 days postadministration of bleomycin in mice, the number of interstitial macrophages (identified as CD64^+^Silgec F^−^) increases, whereas the number of AMs (CD64^+^Siglec F^+^) is reduced. In contrast, during the fibrotic phase (*day 21* postbleomycin administration), the number of IMs decreases, and the number of AMs increases. The same group subsequently developed a lineage tracing system in mice to identify Mo-AMs and tissue-resident alveolar macrophages (TR-AMs) during the development of fibrosis and over the subsequent life span of the animal (37). They used a genetic lineage tracing system to show that Mo-AMs and TR-AMs play distinct roles during the development of lung fibrosis. They showed in the adenoviral TGF-β and bleomycin model of lung fibrosis that deletion of Mo-AM after their recruitment to the lung markedly attenuated the severity of fibrosis, whereas the deletion of TR-AM had no effect on fibrosis severity. These findings are bolstered by transcriptomic profiles of monocyte and macrophage populations over the course of bleomycin-induced fibrosis, which reveals substantial differences in gene expression between Mo-AMs and TR-AMs over the course of lung fibrosis. The authors also performed transcriptomic profiling of flow-sorted monocyte and macrophage populations from lung homogenates, and their results suggest that monocyte to AM differentiation represents a continuous downregulation of genes typically expressed in monocytes and upregulation of genes expressed in AMs. Taken together, their findings suggest that circulating monocytes transition to interstitial macrophages and Mo-AMs.

Monocytes contribute to both the adaptive and innate arms of the immune system. While they can initiate inflammatory responses upon injury, they also participate in normal and aberrant repair processes of injured tissue. For example, a subset of monocytes that displays granulocyte characteristics has been shown to participate in murine fibrosis. These Ceacam1^+^Msr1^+^Ly6C^−^F4/80^−^Mac1^+^ monocytes, named SatM (segregated-nucleus-containing atypical monocytes), are regulated by CCAAT/enhancer binding protein β (C/EBPβ), and C/EBPβ knockout mice do not develop lung fibrosis following bleomycin treatment. However, the adoptive transfer of SatM cells into C/EBPβ knockout mice restored fibrosis susceptibility (68). In addition, depletion of circulating monocytes during the progressive fibrotic phase has been shown to strikingly reduce the degree of fibrosis following lung injury in the bleomycin model of lung fibrosis. In particular, the depletion of a subset of circulating monocytes called Ly6C^hi^ was followed by a reduction of lung macrophage populations, including M2 macrophages (69).

### From Circulating Monocytes to Profibrotic Macrophages

Previous studies reported that the lungs of PF patients and mice exposed to bleomycin are infiltrated by macrophages, which are essential for disease development and progression (70–73). This mechanism seems to be regulated by fibroblast sensing and control of macrophage populations through YAP-mediated mechanotransduction signaling (74–76). Reyfman et al. (77) performed single-cell RNA sequencing (scRNA-seq) on normal and fibrotic (including IPF) lung explants and identified four distinct macrophage clusters. Two of these clusters originated largely from patients with fibrosis and displayed increased expression of fibrotic genes such as MARCK5, IL1RA, PLA2G7, matrix metalloproteinase 9 (MMP9), SPP1, and CHI3L1, which were not detected in normal tissues. Subsequent scRNA-seq studies identified three discrete macrophage subpopulations, which were all present in normal and fibrotic lungs. One had markers of monocyte-derived macrophages, and the other two were resident-like macrophage subpopulations that either expressed high FABP4 and INHBA (FABP4^hi^) or high SPP1 and MERTK (SPP1^hi^) (78). The presence of SPP1^hi^ profibrotic macrophages was confirmed by Adams and colleagues (20). Another relevant finding is the identification of CX3CR1^+^ platelet-derived growth factor (PDGF)-AA^+^ transitional macrophages by scRNA-seq in fibrotic lungs from bleomycin-treated mice. These monocyte-derived, disease-associated macrophages transitioned to AMs and were localized to the fibrotic niche and exerted profibrotic effects, notably via secretion of PDGF-AA, a mitogen necessary for alteration of fibroblast phenotype. Ablation of CX3CR1^+^PDGF-AA^+^ macrophages protected mice from lung fibrosis (79). Interestingly, the authors report an increased expression of CX3CR1 in human fibrotic lungs and found that PDGF-AA^+^ macrophages were associated with fibrotic regions in the lungs (79). In a recent study, Ayaub et al. (80) identified an expanded population of macrophages (IPFeMΦ) in IPF biopsies using a combined scRNA-seq/mass cytometry (CyTOF)/flow cytometry approach. IPFeMΦ expressed a profibrotic signature compared to chronic obstructive pulmonary disease and healthy lung macrophages. The authors reported that these CD84^++^CD36^++^ macrophages displayed a hybrid transitional state between AMs and IMs with M2-like features and specifically expressed Mo-AM markers such as CD14 and APOE, supporting an origin from circulating monocytes (Fig. 1).

### Molecular Mechanisms Controlling Macrophage Activation in PF

**Figure 2.**
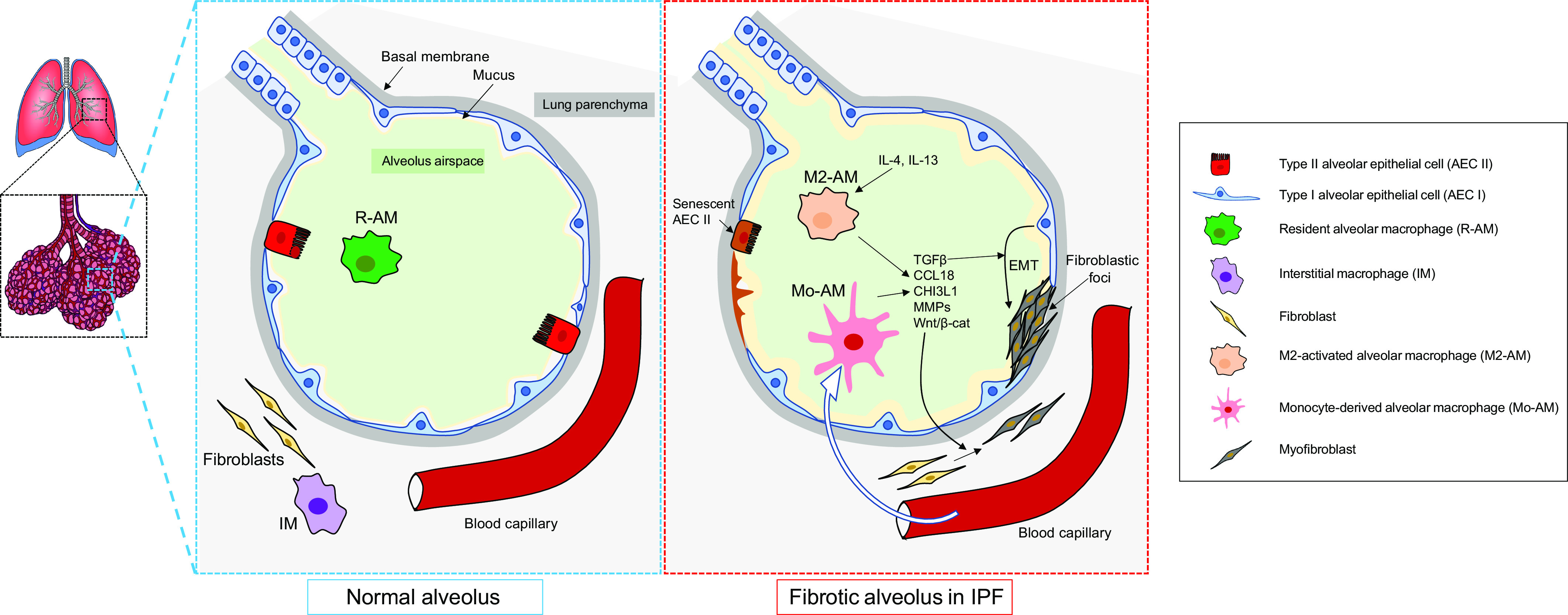
Recruitment and cytokine release by monocyte-derived alveolar macrophages and M2 alveolar macrophages in idiopathic pulmonary fibrosis (IPF). EMT, epithelial-to-mesenchymal transition; TGF-β, transforming growth factor-β. MMPs, matrix metalloproteinases. CCL18, C-C motif chemokine ligand 18.

Both M1 and M2 macrophages participate in the development of lung injury and progression to PF. Lung injury promotes the recruitment of monocyte-derived macrophages that initiate an inflammatory response by producing iNOS and several cytokines (81). Persistent or repeated cycles of alveolar epithelial cell injury promote fibroblast deposition, recruitment of monocyte-derived lung macrophages, and the transition of macrophages to an M2 phenotype. M2 polarization is driven by Th2-secreted cytokines, among which IL-4 is well demonstrated to be an important promoter of the process (Fig. 2). In a bleomycin-induced lung fibrosis model, AMs highly express the Grb2-associated binder proteins Gab1 and Gab2, which mediate IL-4-induced M2 polarization by activating phosphatidylinositol 3-kinase (PI3K)/AKT and JAK1/STAT6 signaling pathways, respectively (82). Interestingly, IL-4/JAK1/STAT6-induced M2 polarization in bleomycin-treated mouse lungs is inhibited by tyrosine phosphatase Shp2, a known antifibrotic regulator in PF (83, 84). Another study demonstrated that signals transmitted from the extracellular matrix via the α_4_β_1_ integrin lead to the activation of Rac2, which in turn regulates alternative macrophage differentiation toward a profibrotic phenotype. Mice deficient in Rac2 are protected against bleomycin-induced PF and display diminished collagen deposition in association with lower expression of alternatively activated profibrotic macrophage markers (85). Mo-AMs with M2-like features are thought to be the main source of TGF-β in lung fibrosis (49, 86), but monocytes and monocyte-derived lung macrophages also release other profibrotic factors such as matrix metalloproteinases that are important for the activation, differentiation, and accumulation of fibroblasts, as well as epithelial-to-mesenchymal transition (87) (Fig. 2). Culture supernatants of AMs from IPF patients induce collagen production in normal lung fibroblasts, a mechanism partly dependent on CCL18 secretion, a marker of M2-like macrophage activation (76). In addition, IL-4-induced M2 macrophages secrete insulin-like growth factor-1 (IGF-1) abundantly, which in turn protects myofibroblast from apoptosis (88). Activated monocytes and macrophages also secrete growth factors such as PDGF and proangiogenic vascular endothelial growth factor, especially the vascular endothelial growth factor (VEGF)-A165b variant, both participating in the fibrotic process and activating signaling pathways that are targeted by Nintedanib (89–93).

Several other mechanisms have been associated with macrophage activation in PF. For example, adenosine, which is generated in excess during cellular stress and damage, is abundant in IPF lungs, and the activation of its receptor, ADORA2B, polarizes macrophages to a fibrotic phenotype (94, 95). Hypoxic conditions lead to hypoxia-inducible factor-1α upregulation, which in turn induces ADORAB2 expression in alternatively activated macrophages (96, 97). Macrophage activation in IPF lung is also related to dysregulated cellular metabolism, a mechanism that includes augmented glycolysis (98). The expression of the glucose transporter GLUT1 is increased in AMs from IPF patients, which enhances glucose intake and subsequent NADPH production (99, 100). NADPH then serves as a substrate of NADPH oxidase (NOX) and peroxynitrite OONO^−^ production, a cytotoxic and tissue-damaging reactive oxygen species (ROS) found elevated in the lungs of IPF patients (101).

Mitochondrial oxidative stress and mitochondrial turnover are also directly linked to AM activation and PF. The expression of the mitochondrial calcium uniporter (MCU), which transports Ca^2+^ into the mitochondrial matrix to modulate metabolism, is increased in IPF AMs (102). MCU-mediated mitochondrial Ca^2+^ influx, as well as ROS and ATP production, modulate macrophage alternative activation to a profibrotic phenotype (103). Additionally, activation of macrophages toward a proinflammatory phenotype can cause impaired mitochondrial respiration and tricarboxylic acid cycle disruption, resulting in the accumulation of endogenous metabolites with potential immunomodulatory roles, such as the antifibrotic itaconate (104, 105). In IPF, decreased levels of itaconate were detected in the patients’ airways, as well as a downregulation of ACOD1, an enzyme that controls the synthesis of itaconate, in airway macrophages (104). In the bleomycin model, *Acod*^−/−^ mice develop persistent fibrosis compared to their WT littermates, and higher expression of fibrosis-related genes was observed in *Acod*^−/−^ airway M2-like macrophages (104).

Endoplasmic reticulum (ER) stress is believed to skew macrophage polarization toward either an M1 or M2 phenotype depending on the pathophysiological context. C/EBP homologous protein (CHOP), a marker of ER, is upregulated in lung macrophages of IPF patients and promotes an M2 program in macrophages through STAT6/PPARγ signaling (71). Iron metabolism has also been associated with macrophage activation during fibrosis. For example, Allden et al. (106) reported the expansion of a transferrin (CD71)-defective AM population in bronchoalveolar lavage (BAL) fluid and lung tissue from IPF patients with progressive fibrotic disease. CD71, which binds most of the free iron in the blood, has been described as a marker of mature macrophages. In IPF, CD71^−^ AMs display functional and phenotypic immaturity and express profibrotic factors such as metalloproteases (MMP)-2 and -9 and plasminogen activator inhibitor 1 (PAI-1), a well-characterized TGF-β transcriptional target. Taken together, these data show the relevance and mechanistic implications of activated lung macrophages and their role in lung fibrosis.

### How Do Macrophages Drive the Fibrotic Process?

Multiple studies have demonstrated the importance of M2-like Mo-AMs in lung fibrosis (107). In mice, the inhibition of M2 polarization protects mice from bleomycin-induced PF (108). In the same murine PF model, depletion and adoptive transfer of Mo-AMs protect or expose animals to PF, respectively (37, 68). However, how these cells precisely induce fibrosis in IPF needs to be addressed. Mo-AMs with M2-like features are thought to be the main source of TGF-β in PF (86, 109), but lung macrophages also release other profibrotic factors that are important for the activation, differentiation, and accumulation of fibroblasts, as well as epithelial-to-mesenchymal transition (EMT) (110) (Fig. 2).

### Macrophages and the Extracellular Matrix

The extracellular matrix (ECM) plays a crucial role in regulating various cellular processes such as adhesion, migration, cell cycle, metabolism, and differentiation. During fibrosis, various enzymes including matrix metalloproteases (MMPs) are responsible for ECM degradation, leading to the formation of fibrotic niches (111). In IPF lungs, high levels of MMPs as well as tissue inhibitors of MMPs (TIMPs) are secreted by macrophages and result in dysregulated collagen turnover and abnormal matrix deposition (112). MMPs increase active TGF-β1 release from the ECM, which in turn regulates ECM deposition and degradation. In addition, overexpression of TGF-β1 has been found to reduce the antifibrotic activity of MMP1 and to induce the expression of tissue inhibitor of metalloproteinase 1 (TIMP1), resulting in an overall increase in ECM deposition. On the other hand, the profibrotic MMP7 has been found to be abundantly expressed in IPF patients and is localized on the surface of AMs (113). Various other macrophage-secreted MMPs have been found to contribute to or to resolve PF and macrophage polarization, including MMP-2, -7, -12, -13, -14, and -28 (114). In a recent article, Adams et al. (20) used scRNA-seq to establish a single-cell atlas of IPF, which uncovered the existence of a profibrotic IPF macrophage archetype among 18 distinct varieties of immune cells. As previously reported, the authors observed an increase in profibrotic and ECM remodeling genes such as SPP1, CHI3L1, CTSK, SPARC (osteonectin), GPC4, PALLD, and MMP9 (20, 78, 115). The metalloprotease MMP9, also called gelatinase B, is predominantly expressed by AMs, and its active form is abundant in BAL fluid from patients with rapidly progressive forms of IPF (116, 117). In addition, blood monocytes and lung macrophages are key cell types contributing to the elevated MMP-8 levels in IPF lungs, and macrophages in areas of mild as well as severe fibrosis robustly express MMP-8 (118). Culture supernatants of AMs from IPF patients induce collagen production in normal lung fibroblasts, a mechanism partly dependent on CCL18 secretion, a marker of M2-like macrophage activation (76). In addition, IL-4-induced M2 macrophages secrete IGF-1 abundantly, which in turn protects ECM-producing myofibroblast from apoptosis (88). M2-like macrophages secrete growth factors such as PDGF-AA and proangiogenic VEGF-A, both participating in the fibrotic process and activating signaling pathways that are targeted by Nintedanib, one of the two Food and Drug Administration-approved drugs in the treatment of IPF (93, 119, 120). IL-10, whose levels are found highly increased in lungs from IPF patients, generates a profibrotic Th2 microenvironment that involves M2 macrophage activation and fibrocyte recruitment likely in a CCL2/CCR2-dependent manner (121, 122).

### Oxidative Stress, Abnormal Metabolism, and Cell Senescence

Pulmonary macrophages were found to promote IPF via the secretion of ROS (40). ROS exert a critical role in the activation of TGF-β, which in turn increases ROS production from mitochondria and NOX activation, forming a profibrotic positive feedback loop (123–125). Interestingly, an excessive number of iron-laden macrophages have been observed in IPF and are associated with oxidative stress and ROS production (126, 127). Yet, iron accumulation shapes macrophage polarization through different signaling pathways and is associated with diffuse vascular abnormalities in IPF patients (128–130). In addition, lipid metabolism seems to be involved in AM-induced IPF. Activated M2-AM uses fatty acid oxidation to increase the expression of arginase-1, which catalyzes the synthesis of ornithine from arginine. Ornithine then serves as an essential precursor for collagen during wound healing (131). Furthermore, nitrated fatty acids (NFAs), which are potent agonists of the antifibrotic nuclear receptor PPARγ expressed in AMs, have been shown to reverse bleomycin-induced lung fibrosis in mice. In AMs, NFA treatment upregulated MFG-E8 expression, a glycoprotein that targets collagen for uptake and lysosomal degradation by macrophages. In addition, NFA promotes myofibroblast dedifferentiation (132). Gao and colleagues (73) reported a potentially important role for netrin (NTN)-1-expressing macrophages in PF, including IPF. The authors showed that macrophage-derived NTN-1 remodeled adrenergic nerves and augmented noradrenaline release, thus promoting lung fibrosis. They found an increased number of NTN-1-expressing macrophages and norepinephrine enrichment in IPF lungs and observed that macrophage-specific deletion of Ntn-1-protected mice against bleomycin-induced PF (73).

IPF is an age-related disease involving cellular senescence, telomere shortening, epigenetic modifications, and mitochondrial dysfunction (12, 133). Aging causes phagocytotic cells to lose function, increasing basal innate immune signaling and decreasing cellular defense. In aging alveolar microenvironments, inflammatory factors such as TNF-α, IL-12, IL-1β, IL-6, IL-10, and IFN-γ are released by aging macrophages. Senescence of type II alveolar epithelial cells results in AM activation and p16 induction, promoting lung fibrogenesis (134). Higher levels of profibrotic IL-10 and macrophage migration inhibitory factor, related to telomere shortening and ECM deposition, are both present in fibrotic niches and BAL in IPF patients (135).

## THERAPEUTIC CONSIDERATIONS AND CONCLUDING REMARKS

Monocytes and monocyte-specific genes have been shown to predict IPF mortality and poor disease outcomes; thus targeting these may represent a novel strategy that could improve the survival of patients with IPF and other forms of PF (1, 24, 136). A recent report demonstrated that administration of diphtheria toxin and conditional depletion of CD11b cells in CD11b-diphtheria toxin receptor mice potently suppressed bleomycin-induced PF (137). Antithetic experiments performed by others have shown that myeloid-specific deletion of activating transcription factor 6 alpha led to increases in CD11b^+^ macrophage subpopulations and worse PF in the bleomycin model (138). Targeting several proteins expressed in macrophages has been shown to ameliorate PF in murine models, particularly when liposomal chlodronate is used to deplete Mo-AM and M2 macrophage populations validating the potential of these cells as therapeutic targets in PF (37, 71, 139, 140). Other compounds targeting pathogenic macrophages such as SHP-1 agonists have been shown to alleviate PF in murine models as well (141).

In addition to macrophage depletion, macrophage reprogramming seems to be a novel and promising therapeutic approach that may be applicable to IPF. Recently, Ahangari and colleagues (142) demonstrated that specific genetic ablation of miR-33 in macrophages protected against bleomycin-induced pulmonary fibrosis by improving mitochondrial homeostasis and increasing autophagy while decreasing inflammatory response after bleomycin injury. The authors also demonstrated that a novel model for pharmacological inhibition of miR-33 in macrophages, via administration of anti-miR-33 peptide nucleic acids (PNA-33), showed attenuation of fibrosis in different in vivo and ex vivo mice and human models of PF (142). In addition, a recent report demonstrated that nanoparticles efficiently delivered small-interfering RNA against TGF-β1, targeting de novo profibrotic Mo-AMs and leading to decreased murine lung fibrosis (143). The therapeutic effect of macrophage reprogramming has been shown by others as well. For instance, Zhang and colleagues (53) created a folate-targeted TLR7 agonist (FA-TLR7-54) that selectively accumulated in profibrotic macrophages and suppressed fibrosis-inducing cytokine production. The authors demonstrate that FA-TLR7-54 reprogrammed M2-like fibrosis-inducing macrophages into fibrosis-suppressing macrophages, resulting in dramatic declines in profibrotic cytokine release and measurements of murine PF (53). Others have shown that macrophages loaded with liposomal dexamethasone and delivered to bleomycin-treated mice reduced murine PF (144). Similar results were seen with the pharmacological blockade of M-CSFR signaling, which led to the disappearance of Mo-AMs and ameliorated fibrosis during asbestos-induced fibrosis (145). Taken together, these results demonstrate that monocytes, Mo-AMs, and profibrotic macrophages are not only mechanistically relevant for PF development and progression, but targeting these key immune cells leads to a reduction in murine PF and may result in therapies that could improve the survival of patients with IPF and other forms of PF. A recent study showed that intravenous administration of mesenchymal stem cells ameliorated pulmonary fibrosis in the bleomycin model through inhibition of monocyte differentiation into M2 macrophages (146). This might revive the interest for future clinical trials studying the effect of stem cells in IPF (147, 148). With regards to other clinical trials, recombinant human pentraxin 2, a potent inhibitor of monocyte differentiation, failed to show efficacy in the recent phase 3 trial, despite previous promising results in the phase 2 trial (149–151). Galectin 3 inhibition, which might affect monocytes’ profile as well, has led to promising data so far, and further results from clinical trials are greatly anticipated (151–153). Further future studies aiming at translating the aforementioned findings into clinical trials are required to determine the clinical applicability of targeting monocytes and lung macrophages in PF.

## GRANTS

This work was supported by the Ubben Family Fund (#250392).

## DISCLAIMERS

The funders did not have any role in paper design, data collection, data analysis, interpretation and writing of the paper.

## DISCLOSURES

No conflicts of interest, financial or otherwise, are declared by the authors.

## AUTHOR CONTRIBUTIONS

C.Y.P., T.K., and J.D.H.-M. conceived and designed research; C.Y.P., T.K., and J.D.H.-M. analyzed data; C.Y.P., T.K., and J.D.H.-M. interpreted results of experiments; C.Y.P., T.K., and J.D.H.-M. prepared figures; C.Y.P., T.K., and J.D.H.-M. drafted manuscript; C.Y.P., T.K., and J.D.H.-M. edited and revised manuscript; C.Y.P., T.K., and J.D.H.-M. approved final version of manuscript.
